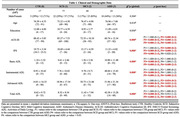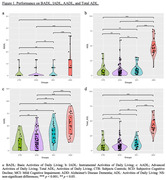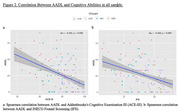# Functional Capacity in Activities of Daily Living in the Alzheimer’s Disease Continuum

**DOI:** 10.1002/alz.088521

**Published:** 2025-01-03

**Authors:** Fernando Henriquez, Carmen Dominguez, Fabrissio Grandi, Cecilia Gonzalez Campo, Patricio Riquelme Contreras, Patricia Lillo, David Martínez‐Pernía, Daniela Thumala, Rodrigo Henriquez, Francisco Aboitiz, Andrea Slachevsky Chonchol

**Affiliations:** ^1^ Neuropsychology and Clinical Neuroscience Laboratory (LANNEC), Physiopathology Department ‐ ICBM, Neuroscience and East Neuroscience Departments, Faculty of Medicine, Universidad de Chile, Santiago Chile; ^2^ Interdisciplinary Center for Neuroscience (NeuroUC) ‐ Laboratory for Cognitive and Evolutionary Neuroscience ‐ Medicine School ‐ Pontificia Universidad Católica de Chile, Santiago Chile; ^3^ Geroscience Center for Brain Health and Metabolism (GERO), Santiago Chile; ^4^ School of Psychology, Universidad de Los Andes, Santiago Chile; ^5^ CONICET, Buenos Aires Argentina; ^6^ Cognitive Neuroscience Center (CNC), Universidad de San Andrés, Buenos Aires, Buenos Aires Argentina; ^7^ Department of Medical Technology. Faculty of Medicine. Universidad de Chile, Santiago Chile; ^8^ Memory and Neuropsychiatric Center (CMYN), Neurology Department, Hospital del Salvador and Faculty of Medicine, Universidad de Chile, Santiago Chile; ^9^ Masters in biological sciences / Neurosciences. Universidad de Valparaíso, Chile., Valparaíso Chile; ^10^ Neurology Unit, Hospital San José, Santiago Chile; ^11^ East Neuroscience Departments, Faculty of Medicine, University of Chile, Santiago Chile; ^12^ Center for Social and Cognitive Neuroscience (CSCN), School of Psychology, Universidad Adolfo Ibáñez, Santiago Chile; ^13^ Interuniversity Center on Healthy Aging, Santiago Chile; ^14^ Department of Psychology, University of Chile, Santiago Chile; ^15^ Neurology Service, Department of Medicine, Clínica Alemana‐Universidad del Desarrollo, Santiago, Chile., Santiago Chile

## Abstract

**Background:**

The most common and prevalent dementia worldwide is Alzheimer’s disease (AD). AD is a continuum composed of Subjective Cognitive Impairment (SCD), Mild Cognitive Impairment (MCI), and Alzheimer’s Disease dementia (ADD) stage. One of the main clinical variables in patients with dementia is performance in functional capacity since its alterations are associated with poor prognosis and disease progression. Functional capacity is measured through activities of daily living (ADL), which are divided into three domains: i) Basic (BADL), ii) Instrumental (IADL), and iii) Advanced (AADL). The study aimed to characterize the performance of the different stages of the AD continuum in the ADL domains and their association with cognitive abilities.

**Method:**

A cross‐sectional study of subjects at different stages of the AD continuum was conducted: Healthy Controls (CTR) (n = 17), SCD (n = 77), MCI (n = 30), and ADD (n = 23), who were matched for age, sex, and education. ADLs were estimated using The Technology‐Activities of Daily Living Questionnaire (T‐ADLQ), which assesses the three domains and a total score. T‐ADLQ performance was compared across groups and correlated with cognitive ability instruments (ACE‐III and IFS).

**Result:**

The results showed that patients with ADD performed worse on the BADL, IADL, and total ADLs compared to the other three groups. There were no significant differences between the CTR, SCD, and MCI on the BADL, IADL, and total ADLs. However, the AADL, in addition to differentiating the ADD patients from the other three groups, also showed differences between CTR and MCI subjects and between SCD and MCI subjects (Table 1 and Figure 1). The correlation study showed that AADL correlated significantly with global cognitive and executive function assessment (Figure 2).

**Conclusion:**

AADL shows progressive functional impairment at different stages of the AD continuum, which is further associated with global cognitive and executive function performances. As one progresses to a more advanced stage of the disease continuum, the performance of ADLs, especially AADLs, worsens, which could indicate a marker of disease progression, allowing for better patient follow‐up.